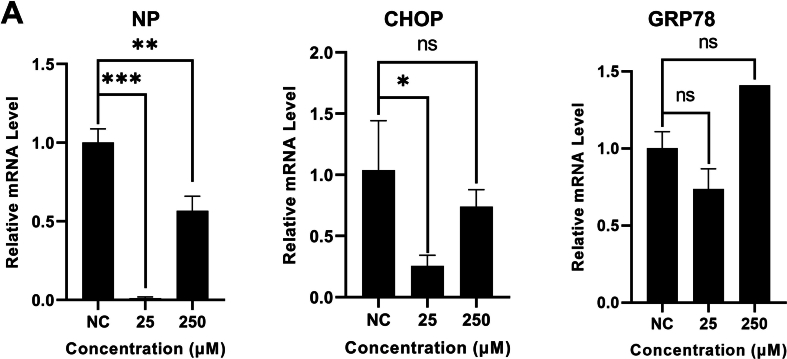# Integrative analysis of the metabolomes and transcriptomes of Ebola virus-infected cells: Uncovering pathways related to hepatic apoptosis

**DOI:** 10.1016/j.gendis.2024.101377

**Published:** 2024-07-16

**Authors:** Chenchen Liu, Zengguo Cao, Cheng Peng, Fangxu Li, Zixi Chen, Xinghai Zhang, Xiaoying Jia, Jinge Zhou, Wenting Mao, Entao Li, Gengfu Xiao, Sandra Chiu

**Affiliations:** a Key Laboratory of Special Pathogens and Biosafety, Wuhan Institute of Virology, Chinese Academy of Sciences, Wuhan, Hubei 430071, China; b University of Chinese Academy of Sciences, Beijing 100190, China; c National Biosafety Laboratory, Chinese Academy of Sciences, Wuhan, Hubei 430020, China; d Shenzhen Key Laboratory of Marine Bioresource and Eco-environmental Science, Shenzhen Engineering Laboratory for Marine Algal Biotechnology, Guangdong Provincial Key Laboratory for Plant Epigenetics, College of Life Sciences and Oceanography, Shenzhen University, Shenzhen, Guangdong 518060, China; e Division of Life Sciences and Medicine, University of Science and Technology of China, Hefei, Anhui 230026, China; f Department of Laboratory Medicine, The First Affiliated Hospital of USTC, Division of Life Sciences and Medicine, University of Science and Technology of China, Hefei, Anhui 230026, China; g Key Laboratory of Anhui Province for Emerging and Reemerging Infectious Diseases, Hefei, Anhui 230026, China

Ebola virus (EBOV) belongs to the Filoviridae family within the genus Ebolavirus and is responsible for the highly lethal hemorrhagic disease known as Ebola virus disease (EVD). The clinical manifestations of EVD include hemorrhagic fever, organ failure, and immune dysfunction.^1^ Liver damage is a significant contributor to mortality in EBOV-infected patients, and cell death plays a pivotal role in this process. Early reports indicated that EBOV infection could trigger multiple forms of cell death, and EBOV-induced hepatocyte apoptosis may be linked to liver injury, as liver conditions are significantly improved in Bim/Bid-deficient mice^2^ after EBOV infection. Choline-related metabolism plays an important role in cell membrane transportation and construction and is a significant indicator of cell injury.^3^ In the context of liver diseases associated with viruses, downstream metabolites of choline, such as fatty acids, serve as key indicators of long-term liver damage.^4^ Viral infection can lead to significant alterations in the host cell's internal environment, resulting in the accumulation of numerous folded and/or misfolded proteins originating from not only cells but also viruses within the endoplasmic reticulum (ER). Further investigation revealed that ER stress can trigger cell apoptosis through multiple signaling pathways.^5^ In summary, we investigated whether choline could attenuate EBOV infection and diminish apoptosis by reducing CHOP (C/EBP homologous protein) gene expression, revealing a novel mechanism by which EBOV induces host apoptosis.

To profile the transcriptome of EBOV-infected HuH-7 cells infected with EBOV at a multiplicity of infection of 1, the cells were harvested each day, and the metabolite samples were harvested at 96 h post-infection (h.p.i.) (Fig. S1A; Fig. 1A–C). Based on the growth curve (Fig. S1B), the data revealed notable enrichment of genes with differential expression at 96 h.p.i., while only 163 and 846 differentially expressed genes were detected at 24 and 48 h.p.i., respectively (Fig. S1C). This observation was reflected in the Venn diagram (Fig. S1D). To explore which cellular functions were persistently activated or inactivated during EBOG infection, we conducted a KEGG enrichment analysis of the genes in the red (constantly down-regulated) and green (constantly up-regulated) clusters (Fig. 1A; Fig. S1E). Notably, the red cluster showed significant enrichment in the fatty acid degradation and fatty acid digestion and absorption pathways, indicating that these two pathways might be related to hepatocyte injury caused by EBOV infection (Fig. S1F). In addition, proteins involved in the ER pathway were enriched in the green cluster (Fig. 1A; Fig. S1G).

**Figure 1:**
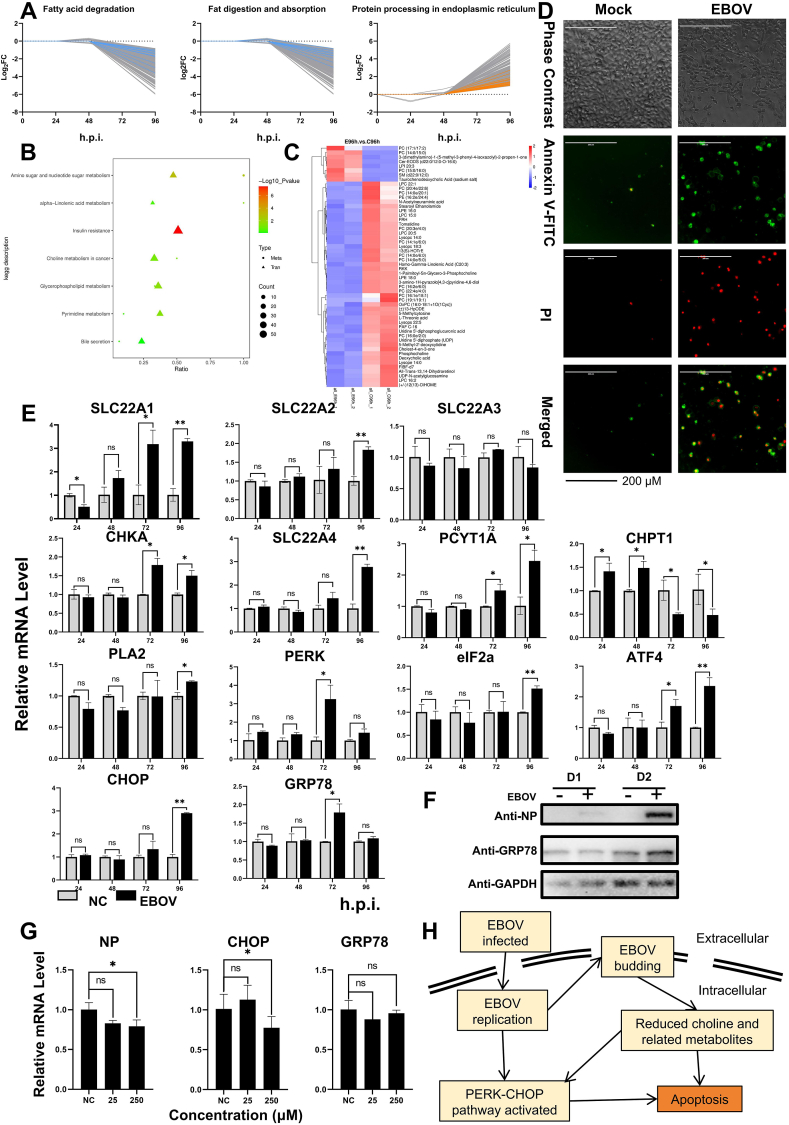
Choline and related metabolites are reduced, and activated endoplasmic reticulum stress induces apoptosis through EBOV infection. **(A)** The gene sets associated with fatty acid degradation and the fatty acid digestion and absorption pathway exhibited a consistent down-regulation trend. The blue line represents the differentially expressed genes enriched in the pathway. The gene sets associated with protein processing in the endoplasmic reticulum pathway exhibited a consistent up-regulation trend. The orange line represents the differentially expressed genes enriched in the pathway. **(B)** Combined KEGG enrichment analysis of metabolic and transcriptomic data revealed significant enrichment of the choline metabolism pathway. **(C)** The heatmap showing the clustergram of differentially abundant metabolites. The red indicates up-regulation, and the blue indicates down-regulation. **(D)** HuH-7 cells exhibited numerous apoptotic events at 48 h.p.i., with both early apoptosis (only green) and late apoptosis (red and green) observed. Apoptotic cell membranes were stained with annexin V-FITC (green), while late apoptotic cell nuclei were stained with propidium iodide (red). **(E)** The line graphs depicting the quantitative PCR results for the genes mentioned in the manuscript at various time points during infection. ∗*P* < 0.05, ∗∗*P* < 0.01. **(F)** The expression of the GRP78 protein increased from 24 h.p.i. to 72 h.p.i., with "miners" representing no infection samples and "plus" representing EBOV infection samples. **(G)** The line graphs illustrating the results in HuH-7 cells. ∗*P* < 0.05. **(H)** Schematic diagram of the EBOV-induced apoptosis pathway. ATF4, activating transcription factor 4; CHKA, choline kinase alpha; CHOP, C/EBP homologous protein; CHPT1, choline phosphotransferase 1; EBOV, Ebola virus; h.p.i., hours post-infection; eIF2α, eukaryotic initiation factor 2α; GRP78, glucose-regulated protein 78; PCYT1A, phosphate cytidylyltransferase 1A; PERK, protein kinase RNA-like ER kinase; PLA2, phospholipase A2; SLC22A1/2/3/4, solute carrier family 22 member 1/2/3/4.

According to the transcriptomic analysis of EBOV infection, fatty acid metabolism clearly decreased, and metabolomic analysis (Fig. 1C; Fig. S2A–C) revealed the same conclusion, namely, lipids and lipid-like molecules are the primary metabolites affected by EBOV infection. Furthermore, the choline metabolism pathway was enriched according to the metabolomic and transcriptomic enrichment KEGG analyses (Fig. 1B; Fig. S2D, E).

Choline is present in both extracellular and intracellular environments, with only a limited amount produced intracellularly. Choline metabolism includes related metabolites, transporters, and enzymes. The metabolic analysis revealed a significant decrease in choline and phosphocholine (Fig. S3A–C). Additionally, the total phosphatidylcholine content markedly decreased (Fig. S3A, D). The choline transporter family includes solute carrier family 57 (SLC57), SCL family 44 member 1/2/3 (SLC44A1/2/3), and SCL family 22 member 1/2/3/4 (SLC22A1/2/3/4). The results indicated an increasing trend in the expression of SLC22A family genes, except for SLC22A3 (Fig. 1E). These findings were in concordance with the transcriptome analysis results (Fig. S3A). Choline metabolism enzymes play a crucial role in transforming choline into downstream metabolites. Choline kinase alpha (CHKA) and phosphate cytidylyltransferase 1A (PCYT1A) were significantly up-regulated at 72 and 96 h.p.i (Fig. 1E). Additionally, phospholipase A2 (PLA2) gene expression was significantly up-regulated at 96 h.p.i. This phenomenon suggested that choline metabolism was abnormal and that the expression of related enzymes was disrupted, which affected the whole metabolic process. The reduced transcript levels of choline phosphotransferase 1 (CHPT1) at 72 h.p.i. enhanced the reduction in phosphatidylcholines during late infection (Fig. 1E).

Choline and phosphatidylcholine can interconvert intracellularly, with phosphatidylcholine being the primary source of choline. Previous studies have shown that choline deficiency can induce apoptosis in host cells. We observed apoptosis at 48 h.p.i (Fig. 1D), and a significant number of HuH-7 cells underwent both early and late apoptosis. Based on these findings, we hypothesize that EBOV induces choline deficiency, subsequently leading to apoptosis.

Recent reports have shown that ER stress induced by viral infection leads to apoptosis. Our transcriptomic analysis revealed a noteworthy up-regulation of genes associated with the protein processing ER pathway at the mRNA level (Fig. S4A, B). The upstream genes glucose-regulated protein 78 (GRP78) and protein kinase RNA-like ER kinase (PERK) were significantly up-regulated at 72 h.p.i., while the downstream genes eukaryotic initiation factor 2α (eIF2α), activating transcription factor 4 (ATF4), and CHOP were significantly up-regulated at 96 h.p.i (Fig. 1E, F). We considered this phenomenon to be normal in hepatocytes; therefore, we used HePG2 as a model and investigated whether the upstream genes GRP78 (Fig. S4C) and PERK (Fig. S4D) were significantly up-regulated after 72 h.p.i., and the trend in gene expression was constantly increasing. As mentioned above, we observed host apoptosis at 48 h.p.i., and the apoptosis-modulating factor PERK CHOP was significantly up-regulated after 72 h.p.i., which indicated that EBOV-induced ER stress could also lead to host apoptosis.

Choline, which has a reported cytosolic concentration of approximately 25 μM, plays a vital role in human physiology. To further elucidate the function of choline during EBOV infection, we treated HuH-7 cells with 25 μM and 250 μM choline at 2 h.p.i. Notably, 250 μM choline significantly reduced the intracellular EBOV RNA level at 96 h.p.i., suggesting the potential of choline to inhibit EBOV infection (Fig. 1G). Interestingly, 25 μM choline significantly decreased the intracellular EBOV RNA level at 96 h.p.i. in HepG2 cells. These findings suggest that the required choline concentration may vary among different cell types (Fig. S5A).

Considering that both choline and its related metabolites are reduced and that ER stress can induce apoptosis, the mechanism through which choline induces apoptosis remains unclear. To investigate the relationship between the reduction in choline and ER stress, we examined the mRNA expression of CHOP and GRP78 after the addition of different concentrations of choline for 96 h.p.i. Surprisingly, choline significantly reduced CHOP gene expression but did not affect GRP78 gene expression during EBOV infection in either HuH-7 cells or HepG2 cells (Fig. 1G; Fig. S5A). These findings revealed that choline can mitigate apoptosis in hepatocytes by decreasing CHOP mRNA expression without affecting GRP78 gene expression.

Early reports suggest that reducing apoptosis in the liver can mitigate damage caused by EBOV infection. In our study, after lowering CHOP gene expression by adding choline to infected hepatocytes, we observed a concurrent reduction in EBOV infection. This suggests that reducing EBOV-induced apoptosis may inhibit EBOV infection. Furthermore, choline, as a potential inhibitor of EBOV and a specific liver repair factor, could serve as a supplementary treatment alongside the other drugs.

In summary, our study established a novel EBOV-infected hepatocyte model *in vitro*, providing insights into the intricate mechanisms of EBOV-induced cell apoptosis and revealing potential implications for therapeutic interventions. We observed that EBOV infection diminished choline and its related metabolites and that choline deficiency led to apoptosis in infected hepatocytes (Fig. 1H). Finally, our research highlights the interplay between choline and ER stress in the context of apoptosis, underscoring the potential of choline as a promising supplement to EBOV.

## Funding

This work was jointly supported by the Strategic Priority Research Program of the Chinese Academy of Sciences (No. XDB0490000), the National Natural Science Foundation of China (No. 82202521), and the China Postdoctoral Science Foundation Funded Project (No. 202122M723345).

## Conflict of interests

The authors declared no competing interests.

## Figures and Tables

**figs1:**
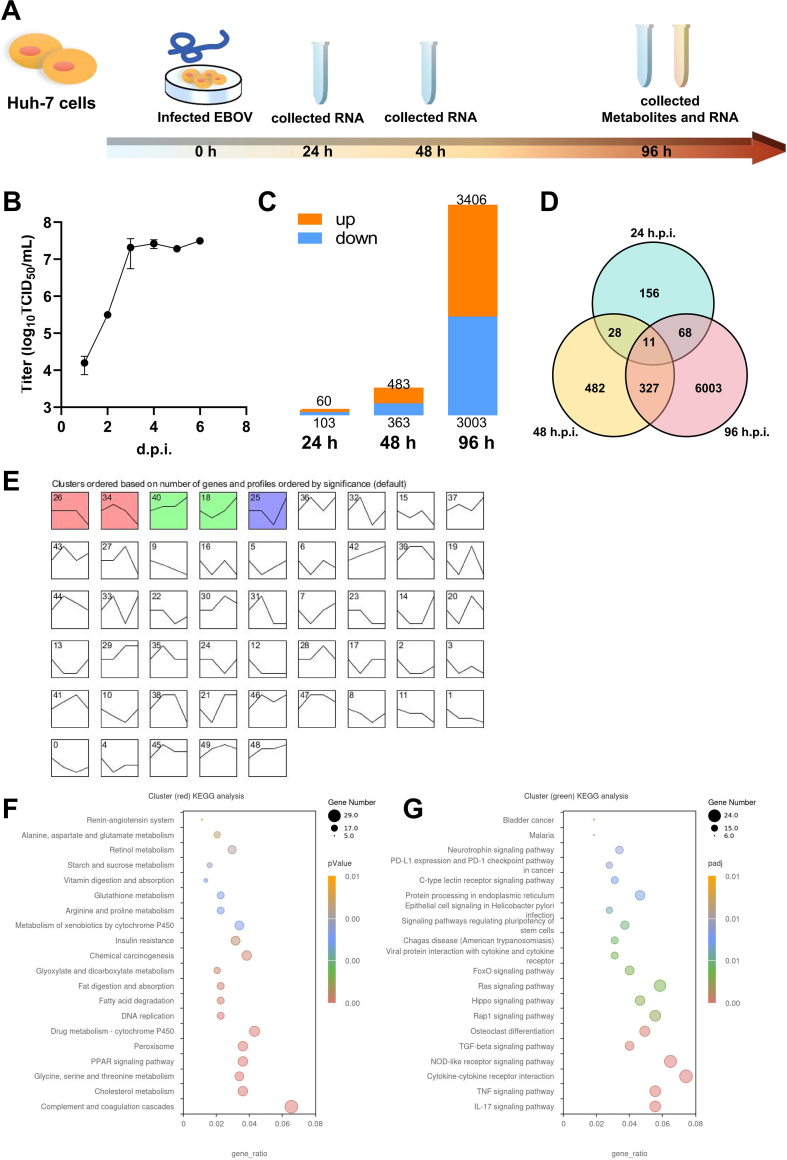


**figs2:**
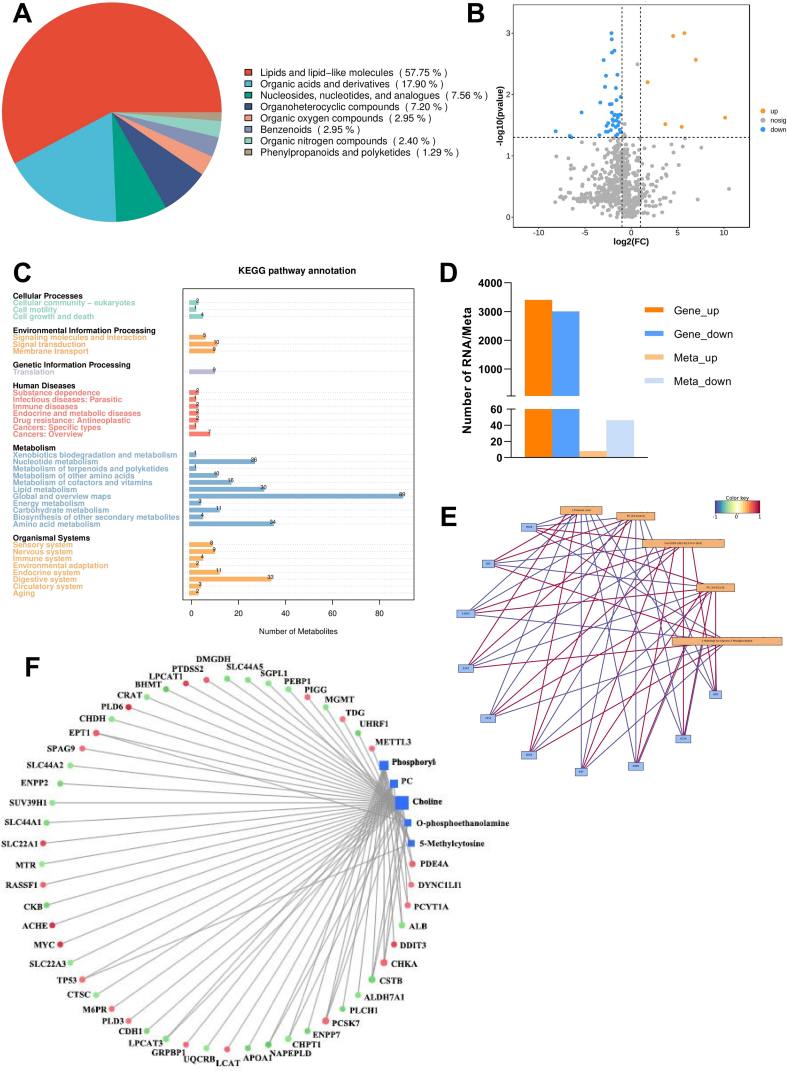


**figs3:**
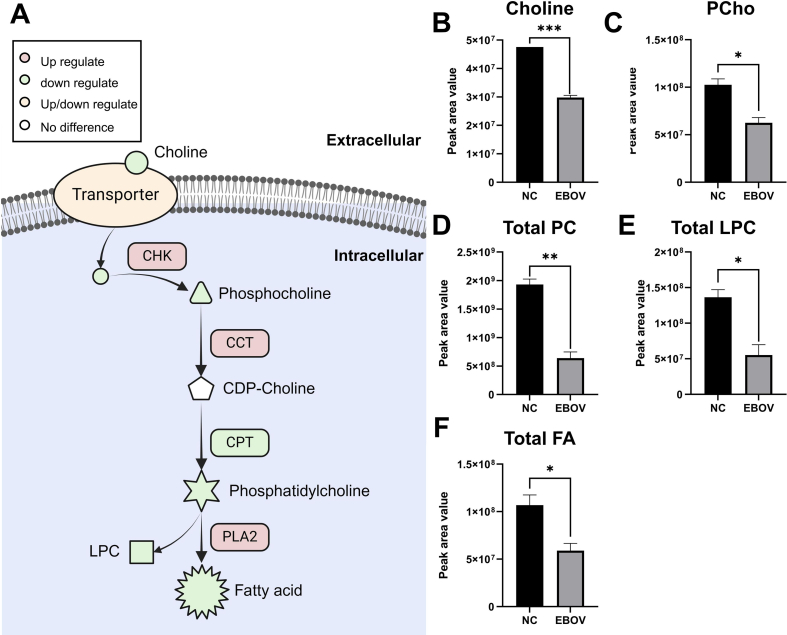


**figs4:**
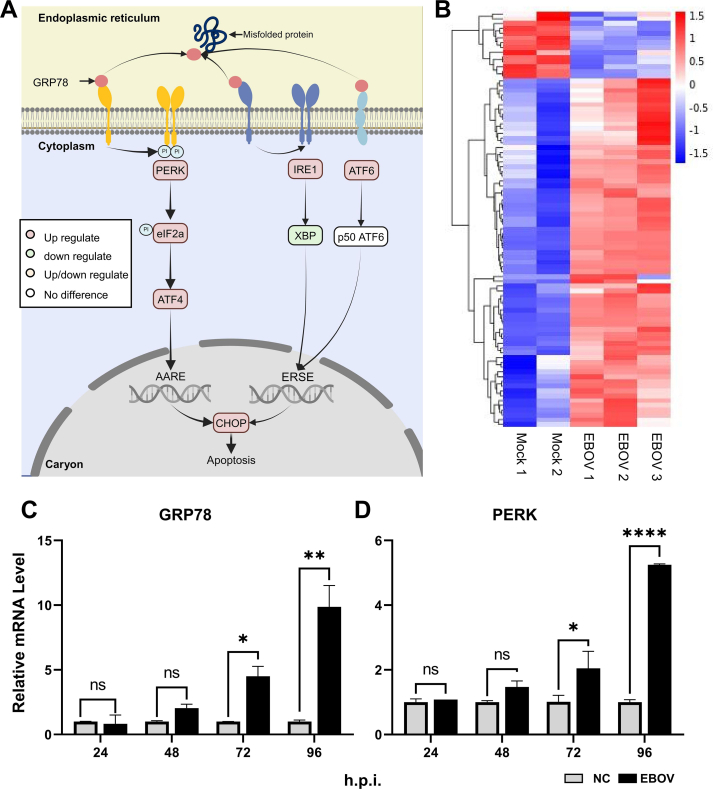


**figs5:**