# Supplementary material for: Integrative analysis of the metabolomes and transcriptomes of Ebola virus-infected cells: Uncovering pathways related to hepatic apoptosis
Source: Genes Dis. 2024 Jul 16;12(2):101377. doi: 10.1016/j.gendis.2024.101377 (PMC11625321; doi:10.1016/j.gendis.2024.101377)
Supplement: Multimedia component 1 [file mmc1.docx]

**Methods and Materials**

Cells and viruses

The HuH-7 cell line was obtained from the American Type Culture Collection. All the cell lines used in the assays were subjected to regular mycoplasma testing. The cells were cultured in Dulbecco’s modified Eagle’s medium (DMEM) supplemented with GlutaMAX, 10% fetal bovine serum (FBS), and 100 U/ml penicillin and streptomycin. Cultures were maintained at 37°C in a humidified environment with 5% CO_2_.

The EBOV strain (Makona-C07, GenBank accession no. KJ660347.2) was stored at the Wuhan Institute of Virology, Chinese Academy of Sciences. The viruses were propagated in Vero-E6 cells, and all the experiments were conducted within a biosafety level 4 (BSL-4) facility.

RNA collection

HuH-7 cells were seeded in 6-well plates at a density of 2×10^5^ cells per well in a total volume of 2 mL per well. The cells were then incubated overnight at 37°C in a 5% CO_2_ environment. Subsequently, HuH-7 cells were infected with EBOV at a multiplicity of infection (MOI) of 1, while the control group remained uninfected.

Each day, cells were collected from each of the three wells using 1 mL of TRIzol per well. To this mixture, 0.4 mL of chloroform was added, and the mixture was gently inverted and allowed to stand at room temperature for 10 minutes. Afterward, the mixture was centrifuged at 12,000 × g for 15 minutes at 4°C. Following centrifugation, 350 μL of isopropyl alcohol was added to the resulting product, which was allowed to stand at room temperature for an additional 10 minutes. Subsequently, the mixture was centrifuged at 12,000 × g for 20 minutes at 4°C. The supernatant was carefully separated, and the RNA was air-dried at room temperature. Finally, 50 μL of ddH_2_O was added to dissolve the RNA, and the RNA was stored at -80°C.

Library preparation for transcriptome sequencing

For RNA sample preparation, total RNA served as the input material. Briefly, the process involved the following steps: 1) Purification of mRNA from total RNA using magnetic beads attached to poly-T oligos. 2) Fragmentation of mRNA with divalent cations in First Strand Synthesis Reaction Buffer (5X) at an elevated temperature. 3) First-strand cDNA was synthesized using a random hexamer primer and M-MuLV Reverse Transcriptase (RNase H-). 4) Subsequent synthesis of second-strand cDNA was performed using DNA polymerase I and RNase H. 5) Convert the remaining overhangs into blunt ends through exonuclease and polymerase activities. 6) The 3' ends of the DNA fragments were adenylated, followed by ligation of adaptors with a hairpin loop structure to prepare for hybridization. 7) cDNA fragments within the preferred length range of 370~420 bp were selected by purifying library fragments using the AM Pure XP system (Beckman Coulter, Beverly, USA). 8) PCR amplification was performed with Phusion High-Fidelity DNA polymerase, universal PCR primers, and an index (X) primer. 10) Purification of PCR products using the AMPure XP system. 11) Assessment of library quality on the Agilent Bioanalyzer 2100 system.

Clustering and sequencing

The index-coded samples were clustered using a cBot Cluster Generation System with the TruSeq PE Cluster Kit v3-cBot-HS (Illumina) following the manufacturer's instructions. Once the clusters were generated, the library preparations were sequenced on an Illumina NovaSeq platform, resulting in the generation of 150 bp paired-end reads.

Transcriptomic data analysis and statistics

Quality control: In the initial data processing, raw data in fastq format were preprocessed using in-house Perl scripts. This preprocessing step involved the extraction of clean data (clean reads) by eliminating reads that contained adapters, reads with poly-N sequences, and low-quality reads from the raw data set. Simultaneously, quality metrics, including Q20, Q30, and GC content, were calculated for the clean data. All subsequent analyses were conducted using clean data, ensuring high data quality.

Reads mapping to the reference genome: The reference genome and gene model annotation files were acquired directly from the genome website. To facilitate alignment, an index of the reference genome was constructed using HISAT2 v2.0.5. Subsequently, the paired-end clean reads were aligned to the reference genome using HISAT2 v2.0.5. HISAT2 was chosen as the mapping tool due to its ability to generate a splice junction database based on the gene model annotation file. This feature enhances the accuracy of mapping, making it a preferred choice over nonsplice mapping tools.

For quantification of the gene expression levels, Feature Counts software version 1.5.0-p3 was used to determine the number of reads mapped to each gene. Subsequently, the fragments per kilobase of transcript sequence per million base pairs sequenced (FPKM) for each gene were computed. The FPKM value takes into account both the impact of sequencing depth and gene length when determining the read count, making it one of the most widely adopted methods for estimating gene expression levels.

Differential expression analysis: In the context of DESeq2 with biological replicates, we conducted a differential expression analysis involving two conditions or groups, each having two biological replicates. This analysis was executed utilizing the DESeq2R package version 1.20.0. DESeq2 offers statistical procedures for identifying differential expression in digital gene expression data, employing a model based on the negative binomial distribution. The resulting P values were subjected to adjustment using the Benjamini and Hochberg approach to control the false discovery rate. Genes characterized by an adjusted P value less than or equal to 0.05, as identified by DESeq2, were considered to be differentially expressed.

GO and KEGG enrichment analyses of DEGs: We performed Gene Ontology (GO) enrichment analysis of the DEGs using the clusterProfiler R package. This analysis incorporated a correction for gene length bias. GO terms with a corrected P value less than 0.05 were considered to be significantly enriched among the DEGs.

The Kyoto Encyclopedia of Genes and Genomes (KEGG) is a valuable database resource that aids in understanding the higher-level functions and utilities of biological systems, spanning from the molecular level to encompass cells, organisms, and ecosystems. This understanding is derived from large-scale molecular data sets, particularly those generated through genome sequencing and other high-throughput experimental technologies (http://www.genome.jp/kegg/).

To assess the statistical enrichment of DEGs within KEGG pathways, we utilized the clusterProfiler R package.

Gene Set Enrichment Analysis

Gene Set Enrichment Analysis (GSEA) is a computational technique employed to ascertain whether a predefined set of genes exhibits a significant, consistent difference between two distinct biological states. This approach entails ranking genes based on the extent of their differential expression in the two samples. Subsequently, predefined gene sets were assessed to determine whether they were enriched at either the top or bottom of this ranked list. GSEA is particularly effective at detecting subtle changes in gene expression.

For GSEA, we utilized the local version of the GSEA tool, which can be accessed at http://www.broadinstitute.org/gsea/index.jsp. In this analysis, GO and KEGG data sets were independently analyzed.

Intracellular metabolite extraction

In the section related to RNA collection, cell culture was mentioned. Each day, cells were collected from each of the two wells using 2 mL of a 4% paraformaldehyde solution per well. Prior to the metabolomics tests, the samples were freeze-dried[;d and subsequently re-extracted using methyl alcohol. The metabolites were then subjected to ultrasonication for 10 minutes (at 40% efficiency) and centrifuged at maximum speed for 30 minutes at 4°C to remove the supernatant.

UHPLC‒MS/MS analysis

UHPLC-MS/MS analyses were conducted using a Vanquish UHPLC system (Thermo Fisher, Germany) coupled with an Orbitrap Q Exactive TMHF mass spectrometer (Thermo Fisher, Germany) at Novogene Co., Ltd. (Beijing, China). The samples were injected onto a Hypersil Gold column (100×2.1 mm, 1.9 μm) with a 17-minute linear gradient at a flow rate of 0.2 mL/min.

Data processing and metabolite identification

The raw data files produced by UHPLC-MS/MS were subjected to processing using Compound Discoverer 3.1 (CD3.1, Thermo Fisher). This software was utilized for tasks such as peak alignment, peak picking, and quantification of each metabolite.

Metabolomic data analysis and statistics

The annotation of these metabolites was conducted using the following databases: the KEGG database (https://www.genome.jp/kegg/pathway.html), the HMDB (https://hmdb.ca/metabolites), and the LIPID Maps database (http://www.lipidmaps.org/).

To identify differentially abundant metabolites, univariate analysis (t test) was used to determine the statistical significance (p value). Metabolites meeting the following criteria were considered to be differentially expressed: variable importance in projection (VIP) > 1, P value < 0.05, and fold change (FC)>2 or FC<0.5. For the generation of clustering heatmaps, the data were normalized using z scores based on the intensity areas of differentially abundant metabolites. These heatmaps were created using the 'heatmap' package in the R programming language.

The correlation between differentially abundant metabolites was analyzed using the 'cor()' function in R (method=Pearson). The statistical significance of correlations between differentially abundant metabolites was calculated using the 'cor.m test() function in R. A P value < 0.05 was considered to indicate statistical significance. Correlation plots were generated using the 'corr plot' package in R.

The functions of these metabolites and their involvement in metabolic pathways were studied using the KEGG database. To assess the enrichment of metabolic pathways associated with differentially abundant metabolites, the following criteria were applied: when the ratio (x/n > y/N) was satisfied, the metabolic pathway was considered to be enriched. A P value of < 0.05 indicated that a pathway was significantly enriched.

Apoptosis detection

Apoptosis was detected using an Annexin V-FITC Apoptosis Detection Kit (Beyotime, C1602S). The antiviral assay was conducted in a manner similar to that previously described using 24-well plates. At 72 h.p.i., the cell supernatant was removed, and 195 μL of Annexin V-FITC binding buffer was added to each well. The buffer was gently mixed, and 5 μL of Annexin V-FITC and 10 μL of propidium iodide (PI) were subsequently added to the wells. After gentle mixing, the samples were left at room temperature in the dark for 25 minutes. The samples were then observed and stored for up to 1 hour.

Quantification of host cell RNA by qRT‒PCR

To quantify the mRNA levels, standard curves were generated using plasmids containing the full-length nucleotides of different genes. Subsequently, quantitative reverse transcription polymerase chain reaction (qRT–PCR) was conducted using the One-step Primer Script RT‒PCR Kit (Vazyme) on a LightCycler (Bio-Rad). The sequences of the primers used are provided in the supplementary materials (Table 1).

Statistics

The data were analyzed using GraphPad 8 software and are expressed as the mean ± standard deviation (SD). Statistical significance was calculated by Student's two-sided t test; *p < 0.05, **p < 0.01, ***p < 0.001, ****p < 0.0001.

Supplementary Data
