# Supplementary material for: Integrative analysis of the metabolomes and transcriptomes of Ebola virus-infected cells: Uncovering pathways related to hepatic apoptosis
Source: Genes Dis. 2024 Jul 16;12(2):101377. doi: 10.1016/j.gendis.2024.101377 (PMC11625321; doi:10.1016/j.gendis.2024.101377)
Supplement: Multimedia component 2 [file mmc2.docx]

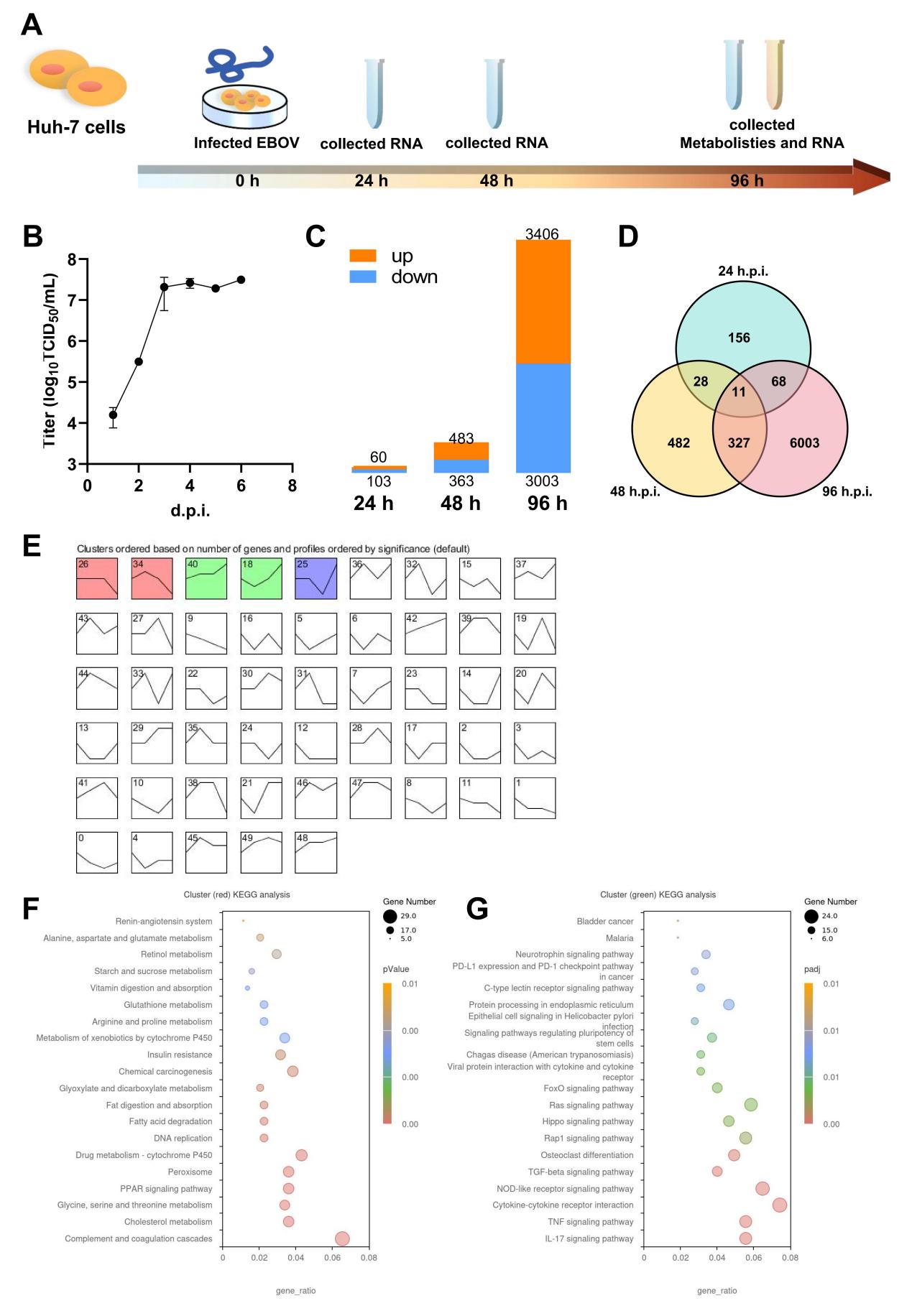


Figure S1 Transcriptomic analysis and spatiotemporal mapping of EBOV infection.(A)EBOV infection model.(B)The growth kinetics of EBOV titration revealed a peak at 72 h.p.i.,indicating the culmination of viral replication,followed by stabilization at 96 h.p.i.(C)At 24 h.p.i.,a total of 163 differential genes were identified,which increased to 846 at 48 h.p.i.The highest number of differential genes,6409,was observed at 96 h.p.i.The bar graph shows the ratio of upregulated(orange)to downregulated(blue)gene sets at various time points post infection.(D)Venn diagram of 24,48,and 96 h.p.i..Elevendifferential genes were constantly involved in EBOV infection.(E)STED analysis of EBOV infection.Spatial-temporal mapping analysis showing clusters with different expression patterns.The red cluster includes profiles 26 and 34,which represent a constantly downregulated trend.The green cluster includes profiles 40 and 18,which represent a constantly increasing trend.The purple cluster includes profile 25.All clusters were statistically significant(p value<0.05).(F)KEGG enrichment analysis of the red cluster revealed significant enrichment in the pathways of fatty acid degradation and fat digestion and absorption.(G)KEGG enrichment analysis of the green cluster demonstrated significant enrichment in the protein processing in the endoplasmic reticulum pathway.


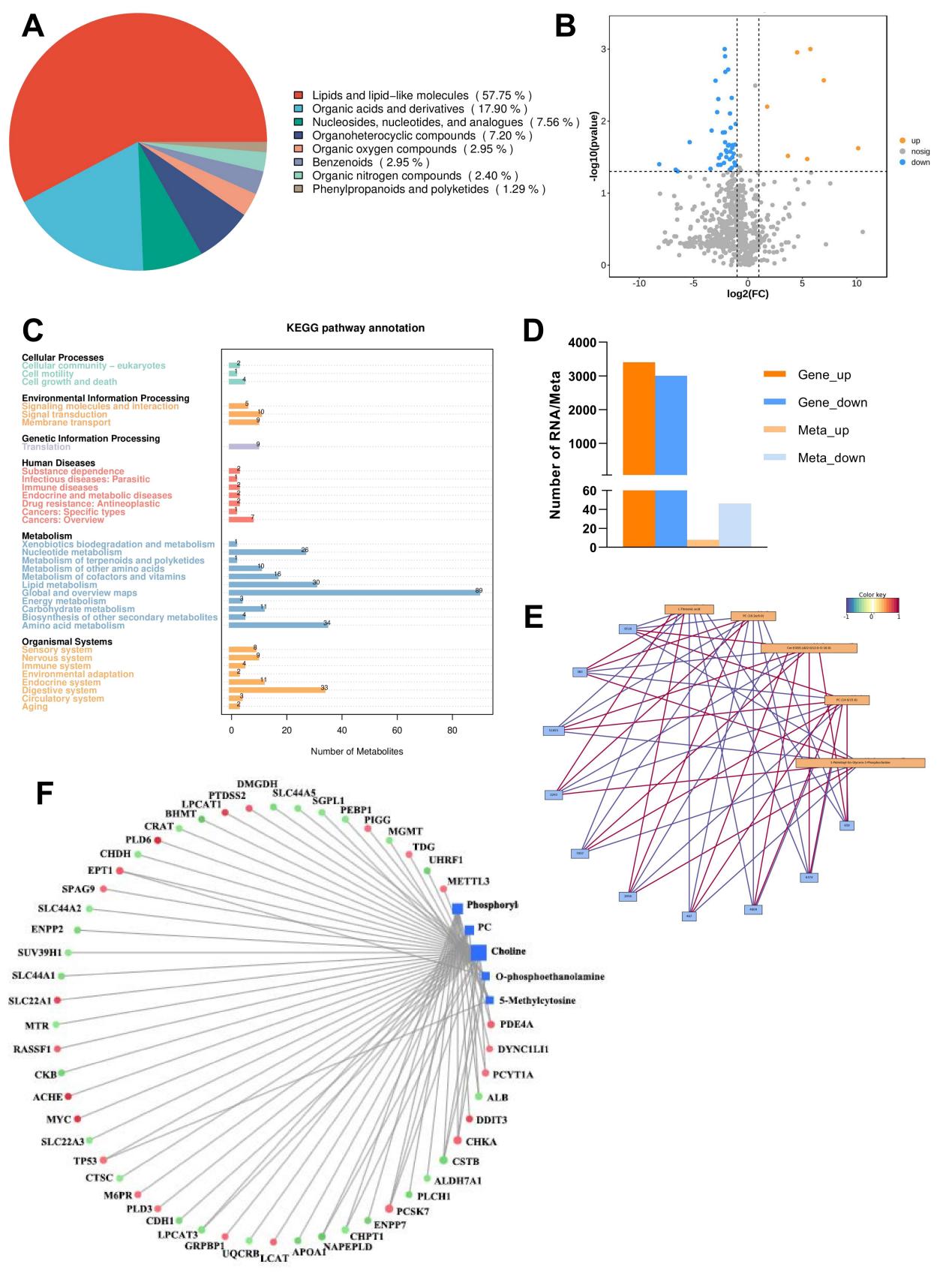


Figure S2 Integrated analysis of metabolic and transcriptomic data.(A)In the analysis of differentially abundant metabolites at 96 h.p.i.,lipids and lipid-like molecules constituted a significant portion of the metabolites,accounting for 57.75%of the total.(B)The volcano diagram illustrates the differentially abundant metabolites at 96 h.p.i.;downregulated metabolites were more predominant than upregulated metabolites.The orange dots represent upregulated metabolites,and the blue dots represent downregulated metabolites,with significance indicated by a threshold of p<0.05.The gray dots represent significantly differentially abundant metabolites(p>0.05).(C)KEGG pathway annotation of differentially abundant metabolites.(D)The number of differential genes was higher than the number of differential metabolites at 96 h.p.i.,making the identification of metabolites more challenging.Bar graph depicting the ratio of upregulated(orange)to downregulated(blue)gene sets and metabolites.(E)Gene metabolism interaction network.


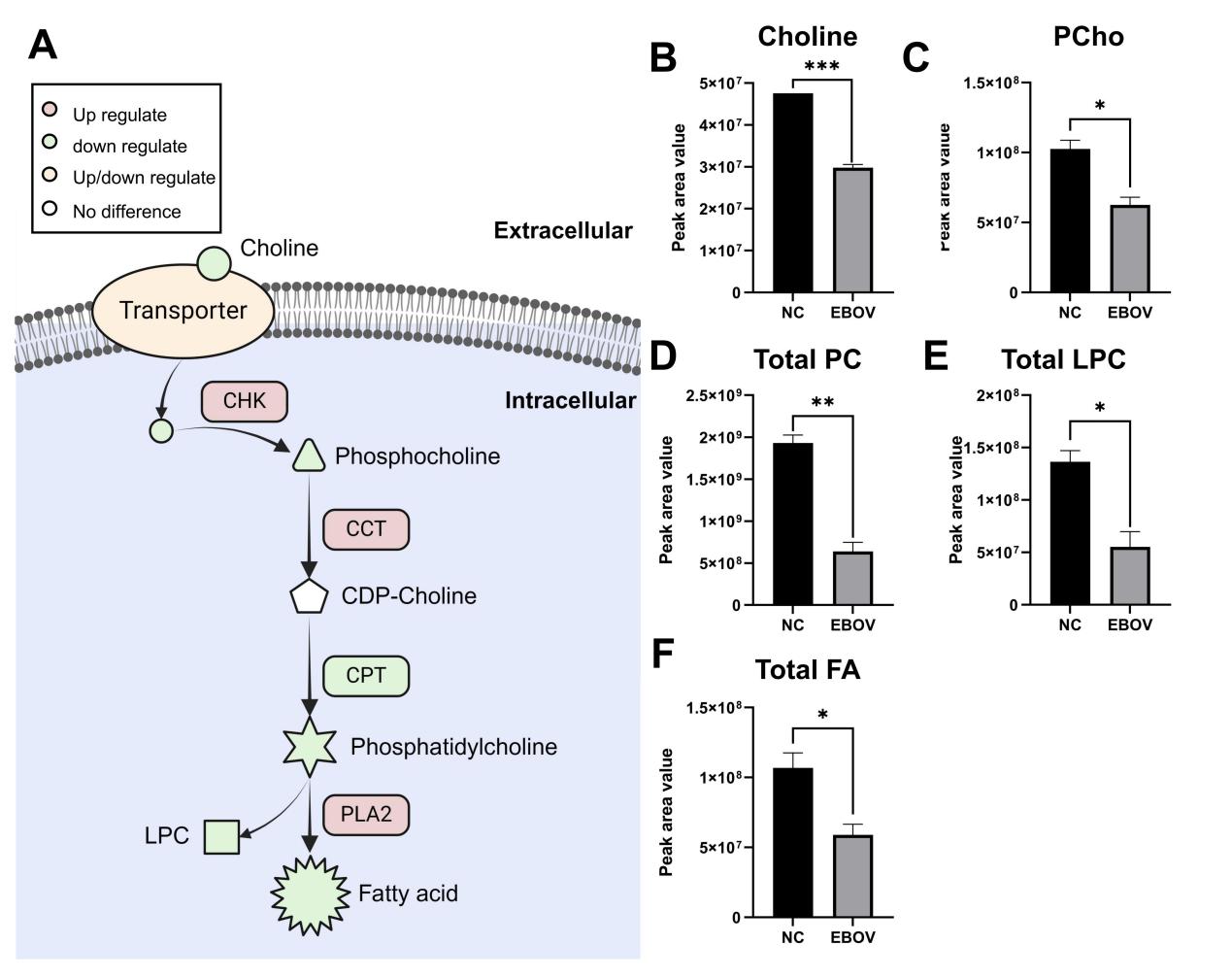


Figure S3 The breakdown of choline metabolism during EBOV infection in HuH-7 cells.(A)The choline metabolism pathway exhibited downregulation of both choline and its downstream metabolites.However,several transforming enzymes were upregulated,possibly attempting to stabilize the pathway by transforming more metabolites.Diagram illustrating the choline metabolism pathway by which more metabolites are transformed.A diagram illustrating the choline metabolism pathway is shown,with red indicating upregulated genes or metabolites,green indicating downregulated genes or metabolites,yellow indicating both upregulated and downregulated genes or metabolites,and white representing no differential genes or metabolites.(B),(C),(D),(E)and(F)Peak area value in the differentially abundant metabolites mentioned in(A).(B)Choline and PC were both downregulated at 96 h.p.i.according to the metabolic analysis.(C)The total PC was obtained by summing the values of all the detected PC metabolites with significant differences;**p<0.01.


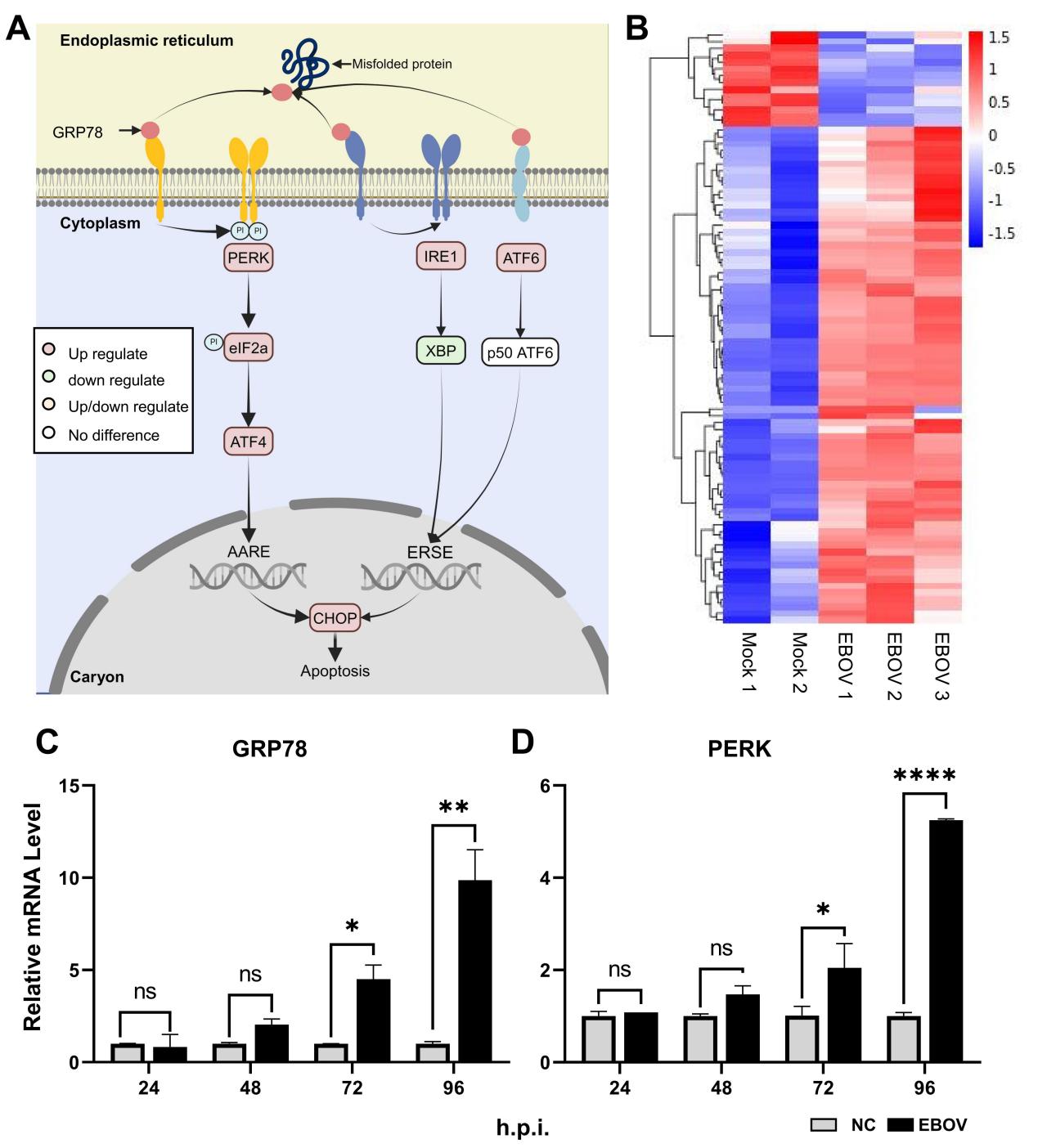


Fig S4 Infection with EBOV induced ER stress and led to cell apoptosis in hepatocyte.(A)Diagram illustrating the ER stress-apoptosis pathway,with red indicating upregulated genes,green indicating downregulated genes,yellow indicating both upregulated and downregulated genes,and white representing genes whose expression did not change.The expression of all the relative genes was upregulated.(B)Heatmap showing the clustergram of differentially expressed genes involved in protein processing in the endoplasmic reticulum.Red indicates upregulation,and blue indicates downregulation.(C)The expression of the GRP78 gene showed a constant increase after 24 h.p.i.during EBOV infection,with a huge significant difference at 72 and 96 h.p.i..The expression of the PERK gene. (D)follow a similar trend in HepG 2 cells,*p<0.05,****p<0.0001.


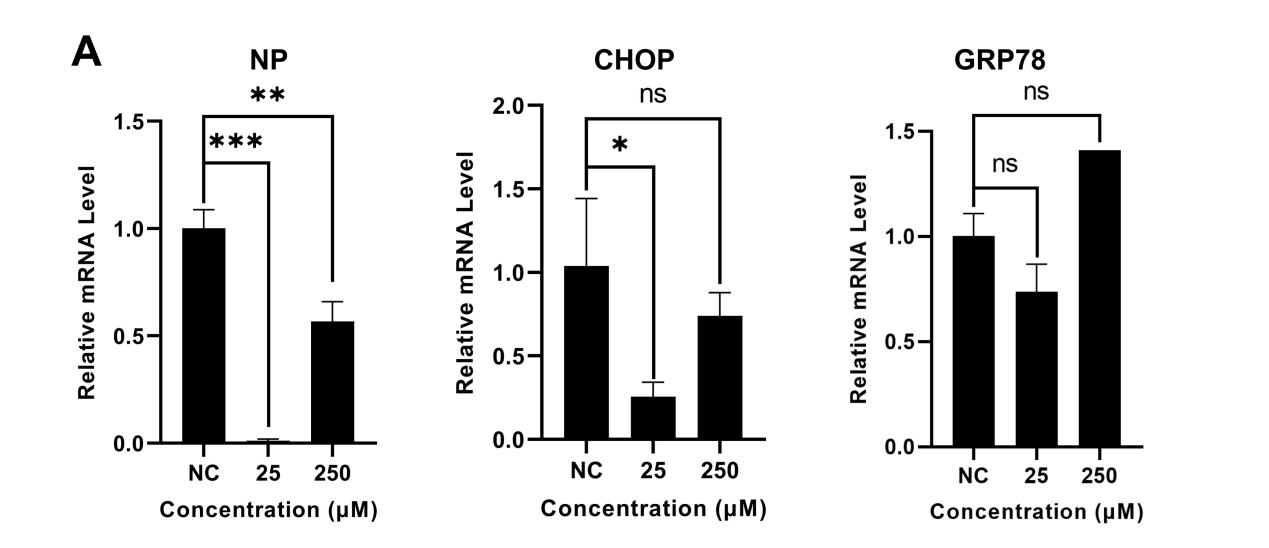


Fig S5 Choline inhibits EBOV infection and decreases apoptosis by modulating CHOP gene expression in HepG2 cells.(A)The line graph illustrates the results in HepG2 cells,revealing no dose-dependent effect of choline on EBOV infection.In HepG2 cells,25μM choline was able to inhibit viral replication,but the effect of 250μM choline was not as effective as that of 25μM choline.Choline(25μM)also reduced CHOP gene expression,but 250μM was not as effective.Choline did not affect GRP78 gene expression in HepG2 cells during EBOV infection (*p<0.05,**p<0.01,***p<0.001).
